# Pulmonary Cryptococcosis and Pulmonary Fibrosis: A Complication of COVID-19 Pneumonia

**DOI:** 10.7759/cureus.35660

**Published:** 2023-03-01

**Authors:** Sangeetha Isaac, Mohammed Afraz Pasha, Shalom Isaac, Evans Kyei-Nimako, Amos Lal

**Affiliations:** 1 Internal Medicine, North Alabama Medical Center, Florence, USA; 2 Critical Care Medicine, Christian Medical College, Vellore, IND; 3 Critical Care Medicine, Mayo Clinic, Rochester, USA

**Keywords:** glucocorticoids, covid-19 ards, covid-19 and pulmonary fibrosis, pulmonary fibrosis, pulmonary cryptococcosis, covid 19

## Abstract

Acute respiratory distress syndrome (ARDS) and pulmonary fibrosis (PF) are increasingly identified as complications of coronavirus disease 2019 (COVID-19) infection, the latter being managed with tapering dose glucocorticoids. Studies have shown improved outcomes with steroid use in this subset of patients; however, the use of high doses of steroids predisposes these patients to develop various complications such as opportunistic infections. The incidence of pulmonary cryptococcosis (PC) in patients with post-COVID-19 PF is not known. Here, we discuss a middle-aged male, with no pulmonary comorbidities, who developed PC secondary to the immunocompromised state from high-dose steroid use for the management of post-COVID-19 PF.

## Introduction

More than two years into the pandemic, clinicians have begun encountering post-coronavirus disease 2019 (COVID-19) sequelae and long-term complications. Acute respiratory distress syndrome (ARDS) and pulmonary fibrosis are emerging as a complication of COVID-19 pneumonia and pulmonary fibrosis (PF) is estimated to be affecting one-third of hospitalized patients, although the exact incidence is not known [1]. Studies have shown rapid and marked improvement in the fibroproliferative phase of ARDS patients managed with high-dose glucocorticoids [2]. However, the use of high-dose glucocorticoids places these patients at a higher risk for opportunistic infections due to their immunocompromised state. Pulmonary cryptococcosis (PC) is an opportunistic invasive mycosis common in immunocompromised patients, presenting as dyspnea and cough [3]. Studies have shown opportunistic infections, such as pulmonary aspergillosis, candida, and pneumocystis pneumonia, in critically ill patients with COVID-19. However, data on the incidence of PC in this subset of patients is sparse [4]. We present a case of pulmonary cryptococcosis complicating post-COVID-19 pulmonary fibrosis intending to highlight the rare post-COVID-19 sequelae.

This case was previously presented as a meeting abstract at the 2021 ACCP Chest annual meeting, October 17-20, 2021 [5]. The abstract was published in the proceeding's journal.

## Case presentation

A 62-year-old, lifelong non-smoker male was admitted to our facility with complaints of shortness of breath and fever for a few days. He had an episode of syncope, prompting his emergency department visit. He denied having a cough. His past medical history was significant for hypertension, chronic back pain, gastroesophageal reflux disorder, benign prostatic hyperplasia, and pulmonary embolism 20 years ago.

An initial evaluation in the emergency department revealed new-onset acute hypoxic respiratory failure secondary to multifocal COVID-19 pneumonia confirmed with COVID-19 polymerase chain reaction (PCR), which was complicated by acute pulmonary embolism involving the right upper and bilateral lower lobe pulmonary arteries. He was admitted to the intensive care unit and bi-level non-invasive positive pressure ventilation (NIPPV) therapy was commenced. He was managed with intravenous remdesivir, ceftriaxone, azithromycin, and a therapeutic dose of enoxaparin. He received intravenous dexamethasone 6 mg daily, which was subsequently increased to once in six hours within 48 hours of admission due to the inability to wean off the fraction of inspired oxygen (FiO2) on bilevel positive airway pressure (BiPAP). Steroids were tapered gradually and discontinued at discharge. At the time of discharge, he was on 4L/min oxygen.

Three weeks after discharge, the patient presented to the emergency department with complaints of worsening shortness of breath even at rest. On presentation, he was afebrile 98.5°F, tachycardic with a heart rate of 114/min, a respiratory rate of 20/min, normotensive with a blood pressure of 126/62 mmHg, and saturating 93% on 6L/min oxygen. He had bilateral crackles on chest auscultation. He did not have any rhonchi or wheeze. The cardiovascular system examination was unremarkable.

**Figure 1:**
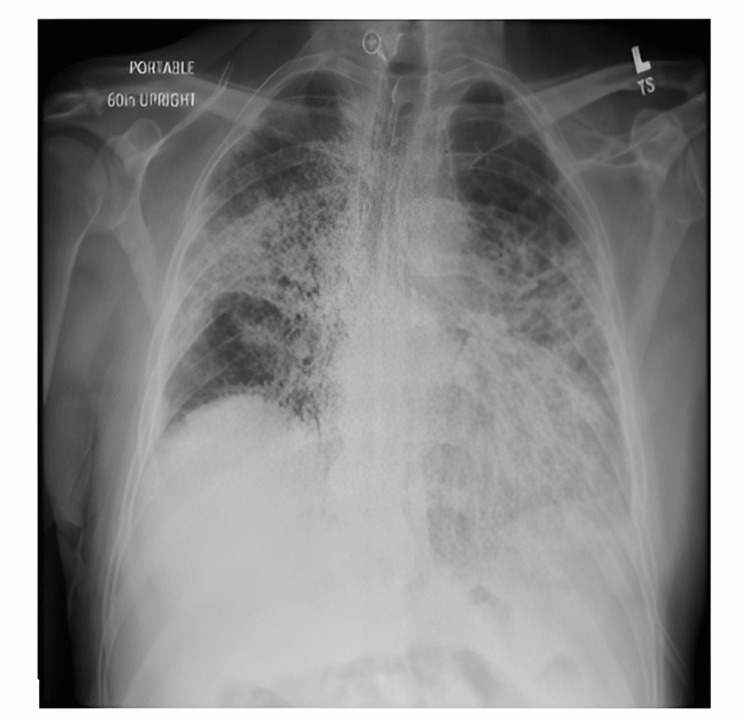
Antero-posterior view of chest X-ray demonstrating multifocal pneumonia

Initial investigations were notable for a white blood cell count of 8,300 cells per cubic ml (normal range- 4,500 to 11,000 WBCs per cubic ml), hemoglobin 10.0 gm/dL (normal range- 14gm/dL-18gm/dL), and platelet count of 212,000 per cubic ml (normal range 150 per cubic ml - 375 per cubic ml). D-dimer was 0.86 (normal range <0.59). Sodium 138 mmol/L (normal range -135 mmol/L- 145 mmol/L ), potassium 4.4 mmol/L (normal range 3.6 mmol/L- 5.2 mmol/L), chloride 96 (normal range 98 mmol/L- 108 mmol/L), bicarbonate more than 40 mmol/L (normal range 21 mmol/L - 32 mmol/L), urea 26 mg/dL (normal range 4 mg/dL- 22 mg/dL), and creatinine 0.9 mg/dL (normal range 0.6 mg/dL - 1.3 mg/dL ). Chest X-ray showed multifocal infiltrates are unchanged (Figure 1). Arterial blood gas on 44% Fio2 showed a normal pH of 7.38, with hypoxia pO2 58 and hypercarbia pCo2 65. COVID-19 PCR remained positive.

**Figure 2:**
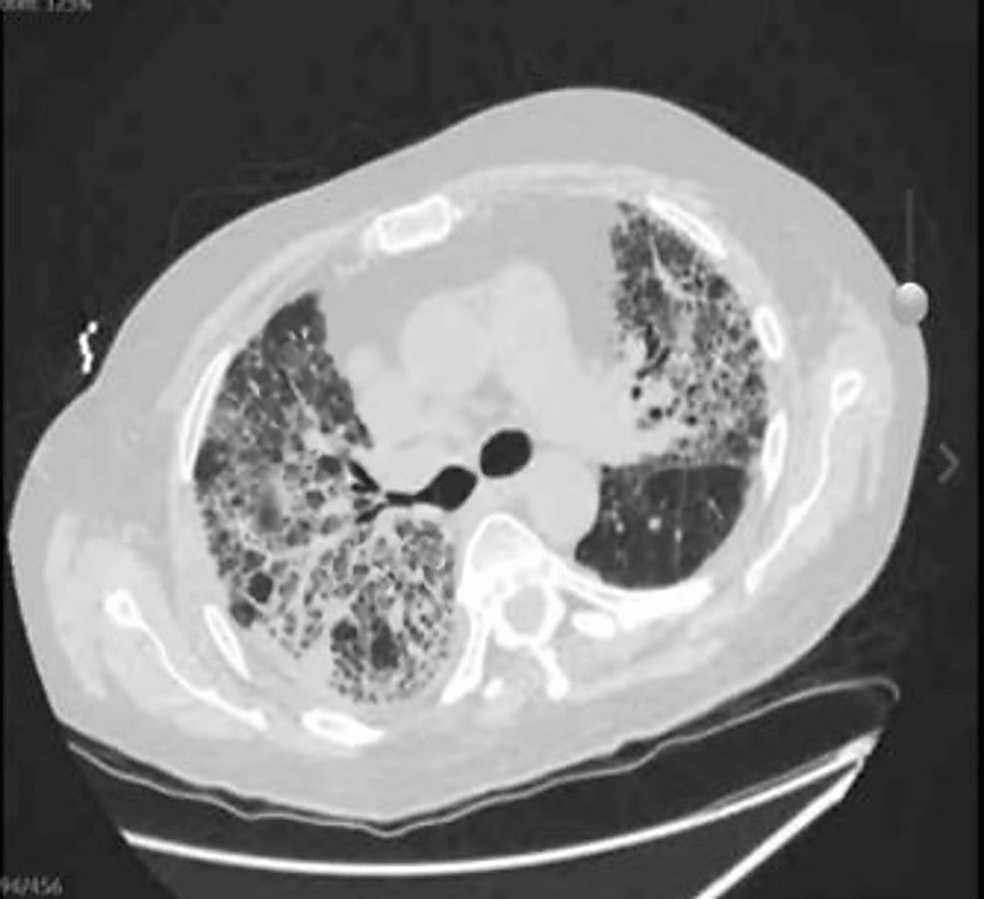
Axial view of CT chest demonstrating severe multifocal pneumonia with interval development of bronchiectasis

The patient was initiated on intravenous linezolid, cefepime, and dexamethasone while continuing apixaban. Chest CT without contrast revealed severe multifocal pneumonia with interval development of bronchiectasis and extensive fibrotic changes (Figure 2). Worsening hypoxia led to the commencement of bi-level non-invasive positive pressure ventilation (NIPPV) therapy. The patient was initiated on intravenous methylprednisolone 125 mg every six hours in view of suspected post-COVID-19 pulmonary fibrosis. One week following the initiation of methylprednisolone, the patient’s oxygen requirement still remained high with difficulty weaning FiO2. Empiric antifungal treatment with voriconazole was started at 200 mg twice daily as a fungal infection was suspected.

Fungal studies and serology were positive for cryptococcal antigen (Table 1) when treatment was changed to intravenous fluconazole. Cerebrospinal fluids analysis was deferred since he had no neurological manifestations. Of note, he did not have any other immunocompromise such as HIV, diabetes mellitus, or end-stage renal disease. Methylprednisolone was continued for a total of two weeks, following which it was tapered and discontinued.

**Table 1:** Serological investigations

Serology:	Result:
Aspergillus antibody	Negative
Blastomyces antibody and urinary antigen	Negative
Histoplasma antibody and antigen	Negative
Coccidiodes antibody and antigen	Negative
Cryptococcal antibody	Negative
Cryptococcal antigen	Reactive Titer >1:8

The patient’s hospitalization was further complicated by critical illness myopathy, atrial fibrillation, flutter, and acute diastolic congestive heart failure with severe pulmonary hypertension, which was appropriately managed simultaneously.

The patient showed marked clinical improvement during the hospitalization and a repeat CT scan done 18 days after starting fluconazole showed interval improvement of multifocal infiltrates. He was advised to continue fluconazole for six months with an interim follow-up with the pulmonary medicine clinic.

## Discussion

Post-COVID-19 manifestations are increasingly reported with evolving clinical data, with only 10.8% of patients having no manifestations [6]. Fatigue was the most common symptom seen in about 72.8%; stroke, pulmonary fibrosis, and myocarditis are some of the less frequently reported complications [1,7]. Dysregulated release of matrix metalloproteinases, epithelial injury, and fibroproliferation are all implicated in the pathogenesis of post-infective pulmonary fibrosis [4]. Vascular dysfunction mediated through vascular endothelial growth factors and cytokines like interleukin-6 and tumor necrosis factor-α has been implicated to play a key role in the transformation of ARDS to fibrosis [4,8].

Several studies have been published studying the appropriate dosing for the treatment of COVID-19 ARDS and the debate about low-dose versus high-dose dexamethasone has so far not reached a consensus [9-12]. Glucocorticoids are given widely to patients developing pulmonary fibrosis post-COVID-19 infection [13]. High-dose steroids used in the management of pulmonary fibrosis pose a serious risk of complications related to immunosuppression and subsequent opportunistic infections such as pulmonary cryptococcosis and pneumocystis pneumonia.

Cases of pulmonary cryptococcosis in immunocompromised patients are emerging with a few case reports published. The risk factor predisposing our patient to the development of pulmonary cryptococcosis was prolonged steroid use, in the setting of post-COVID pulmonary fibrosis. Other risk factors, such as poorly controlled diabetes, cirrhosis, use of immunosuppressive drugs, and malignancy, have been reported [14]. Most patients with pulmonary cryptococcosis present with cough and dyspnea. Diagnosis entails investigations such as cryptococcal antigen detection, bronchoalveolar lavage cultures showing cryptococcus neoformans, or histopathology of the biopsy showing granuloma with encapsulated yeast [15]. CSF microscopy or polymerase chain reaction may be positive in cryptococcal meningitis [16]. Our patient was appropriately managed with fluconazole per the Infectious Diseases Society of America recommendation [17], with which he had significant improvement. While prevention is not always possible, special attention should be given to patients at risk for developing cryptococcosis, for timely identification, and intervention.

## Conclusions

Our patient developed pulmonary cryptococcosis that was managed with fluconazole, which is the first-line treatment, with significant clinical improvement. Our patient did not have CNS manifestations; however, if he had exhibited signs and symptoms concerning for the same, amphotericin or flucytosine would have been more appropriate in his management.
